# Declining Sleep Quality among Nurses: A Population-Based Four-Year Longitudinal Study on the Transition from Nursing Education to Working Life

**DOI:** 10.1371/journal.pone.0014265

**Published:** 2010-12-08

**Authors:** Dan Hasson, Petter Gustavsson

**Affiliations:** 1 Department of Physiology and Pharmacology, Karolinska Institutet, Stockholm, Sweden; 2 Stress Research Institute, Stockholm University, Stockholm, Sweden; 3 Section for Psychology, Department of Clinical Neuroscience, Karolinska Institutet, Stockholm, Sweden; University of Sydney, Australia

## Abstract

**Background:**

Several studies have established impaired sleep is a common problem among nurses. Overworked, fatigued and stressed nurses are at a higher risk of making mistakes that threaten patient safety as well as their own health. The aim of the present study was to longitudinally monitor the development of sleep quality in nurses, starting from the last semester at the university, with three subsequent annual follow-ups once the nurses had entered working life.

**Methodology/Principal Findings:**

Nationwide, longitudinal questionnaire study of nursing students and newly qualified nurses in Sweden. The results imply a continuous decline in sleep quality among nurses during the three years of follow-up, starting from their last semester of nursing education and continuing for three years into their working life. The most pronounced short-term decline in sleep quality seems to occur in the transition between student life and working life.

**Conclusion/Significance:**

This finding is important since it may affect the quality of care and the health of nurses negatively.

## Introduction

Several studies have established that nursing is a stressful profession [1], [2], [3], [4], [5], [6], [7] and stressful working conditions for nurses have been suggested to be influential on sick leave [8], turnover rates and the likelihood of leaving the job [9], [10]. Whereas acute stress is generally adaptive, long-term exposure to stress without sufficient recovery may cause various adverse health consequences and diseases [11], [12], [13], [14], [15], [16]. Impaired recovery seems to be an important mediator between stress and different kinds of ill health and diseases. In fact, both acute and chronic stress has been found to be associated with subjective and objective measures of impaired sleep and recovery [17], [18], [19], [20], [21]. Furthermore, coherent with the statement that nursing is a stressful profession, studies have reported that fatigue due to inadequate or impaired sleep is a common problem among nurses [4], [22], [23], [24]. Overworked, fatigued and stressed nurses are at a higher risk of making mistakes that threaten patient safety [25], [26]. Their condition may also affect levels of sickness absenteeism or job turnover and thereby making it difficult to maintain adequate levels of health care staffing, reducing quality of care and threatening patient outcomes [10], [27], [28]. Shift work is also common among nurses and it has been shown that it can increase prevalence of sleeping problems and chronic fatigue as well as impair recovery [29], [30], [31].

### Trends and patterns of sleep in the general population

American data suggest that since 1910, the average “normal” sleep duration may have decreased from about 9 hours to approximately 7.5 hours today [32], [33]. At present, prevalence of impaired sleep is reported to be common (about 10–30%) in the general population [34], [35], [36], [37], [38], [39], but there is no consensus as to how common [38], [40]. Results from population-based surveys in Sweden indicate that impaired sleep in the general population seems to have become increasingly common during the last 20 years, with a prevalence increasing from 30% in 1986 to 47% in 2001 [41]. Impaired sleep also seems to be frequent in the USA, especially among women. In the recently published 2007 Sleep in America Poll, 60% of the women stated that they only get a good night's sleep a few nights per week or less [42]. As many as 67% of the women experienced sleep problems at least a few nights each week, with 46% experiencing them every night.

### The stress-sleep interaction

Stress and sleep can be described as counterparts that interact and affect each other in various ways. For example, stress increases arousal whereas sleep decreases it. Stress disturbs homeostasis [13], [14], [15] and sleep appears to be important for restoring and maintaining it [34]. Hence, sleep is suggested to be an important “antistress” mediator that counteracts the wear and tear of stress on individuals [34]. Furthermore, it is well-established that stress impairs sleep [18] and stress is also considered to be the main cause of some sleep disorders, e.g. primary insomnia [43]. On the other hand, impaired sleep seems to be a stressor by and of itself, and may lead to stress-related mental ill health such as anxiety and depression [34], [44]. Both long-term stress exposure and impaired sleep is associated with alterations of the hypothalamic-pituitary-adrenal (HPA) axis, which is the main neuroendocrine moderator of the biological stress response [11], [12], [32], [34]. At the same time, stress that is discontinued may increase and improve sleep quality and recovery [18], [34]. Also, following insufficient sleep or sleep deprivation, the length and quality of sleep (decreased fragmentation/awakenings) and the share of deep sleep increases [45].

### The stressful transition between nursing education and working life

For nurses entering working life, taking on a professional role is associated with increased responsibilities, e.g. being accountable and responsible for choices that may affect patients' health and wellbeing adversely. The transition between nursing education and working as a nurse is described as being “massive” or as a “reality shock” [46], [47], [48]. Paralleling these changes are the high levels of individual, environmental, occupational and organizational stress characterizing the work of nurses that have been described in several studies [2], [49], [50]. The transition for nurses from higher education to working life thus involves several types of change and seems to be a major contributing cause of distress and consequently also ill health, on a longer term basis [46], [47], [51], [52], [53], [54], [55]. In view of this, it is important to study how the transition from higher education to working life influences sleep quality in nurses.

### Aims

In summary, previous research indicates high levels of stress and fatigue among nurses. As most studies assessing sleep quality are cross-sectional, there is a need for longitudinal monitoring to evaluate possible changes and patterns over time. Therefore, the aim of the present study was to longitudinally monitor the development of sleep quality in nurses, starting from the last semester at university, with three subsequent follow-ups once the nurses had entered working life.

## Methods

### Ethical approval

The Karolinska Institute ethics committee approved the research project and all participants gave their written informed consent.

### Participants

LANE (Longitudinal Analyses of Nursing Education) is an ongoing nationwide longitudinal project focusing on mapping health and career development among nursing students who become newly qualified nurses in Sweden. The cohort of the present study is based on a total population of all the final semester nursing students in the autumn of 2002.

In all, 1,648 final semester nursing students from 24 universities or university colleges were invited to participate. Of these, 1,114 (67.6%) enrolled in the study. This group constitutes the cohort. Looking at the different universities, it was concluded that some of these were underrepresented or overrepresented in the cohort (χ^2^ = 63.4, p<.001). However, there were no significant age- or sex-related differences between the participants and non-participants.

With regard to the 534 (32.4%) non-participants, the main reason for not being part of the cohort was failure to return the questionnaire. A minor number of non-participants were excluded due to logistic factors such as identification problems, erroneous serial numbers or a clearly expressed unwillingness to participate in the study.

**Figure 1:**
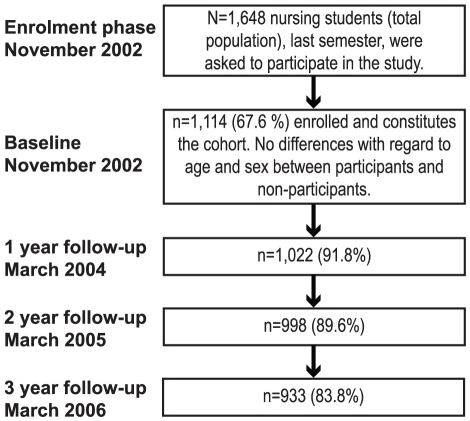
Flow of the participants and questionnaire distribution.

Figure 1 depicts the flow of the participant cohort during the first four years of the project. In total, 866 (78%) individuals participated on all four measurement occasions.

### Questionnaire

A comprehensive questionnaire was sent to all students including some single-items and previously validated measures of socioeconomic status and other demographic, health-related and lifestyle factors. Major parts of this questionnaire were also included at the follow-ups, together with questions on working conditions.

With regard to sleep quality, the participants provided self-ratings along a five-grade response scale with the answer alternatives: *Good* (5), *Pretty good* (4), *Neither good nor poor* (3), *Pretty poor* (2), *Poor* (1). The question has been previously validated [21]. The internal dropout (missing data) for the sleep quality ratings varied between four to nine individuals across all measurement occasions.

The questionnaire was distributed by mail (with two reminders to non-responders) at baseline: the sixth and last semester of the nursing program. The second to fourth follow-up assessments were conducted after one, two and three years in working life (Figure 1).

### Statistical analyses

Initially, three datasets were created from the complete dataset. The first consisted of the 846 participants who provided sleep quality ratings in all four assessments (referred to as “the listwise sample” below). Using the EM (expectation-maximization) algorithm for imputation of missing values, a second dataset including estimates for individuals with missing data was created (n = 1114). The EM uses an algorithm to estimate the means, covariances, and Pearson correlations of quantitative variables by computing expected (E) values conditional on the observed data and the current estimates of the parameters and maximum-likelihood (M) estimates of the parameters based on values that are computed in E calculations. Finally, a dataset including only participants working as nurses on each and every follow-up occasion (n = 554) was compiled. The participants who were excluded from this final dataset were mainly on maternity-leave or committed to advanced nursing education on one or more of the measurement occasions.

A repeated measures ANOVA was conducted to assess possible change in mean sleep quality over time, and effect size was calculated as eta squared statistics. The interpretations were based on established cut-offs [56]. ANCOVAs were then utilized to measure potential interaction between factors such as age groups (divided by quartiles) and sex, as well as factors such as whether or not the nurses were committed to a steady relationship, were living alone (or with parents), had children at home, and whether or not they had previous nursing assistant training, other previous experience in healthcare, or felt their education had prepared them well enough to work as nurses. The covariate in the ANCOVAs was baseline sleep quality.

To rule out possible selection bias, logistic regression models were utilized to predict response vs. non-response at each assessment. Sleep quality from each previous assessment was used together with age and sex as predictors of response vs. non-response at each and every follow-up. No significant effects of these variables were found, which renders the interpretation that there was no serious selection bias. The program SPSS 15.0 for Windows was used for statistical analyses.

## Results

At baseline, there were 986 (89%) women and 120 (11%) men in the study. The age ranged from 21–52 years with a mean age of 30.4±7.4 years. Median age was 28 years and interquartile range was 24–36 years. With regard to other background variables used in the present study at baseline, 723 (66%; valid percent) of the participants were living with partners; 366 (34%) were not. Furthermore, 311 (28%) were living alone or with parents; 466 (43%) were living with children at home; 591 (53%) had previous experience from work within healthcare.

### Mean values

**Figure 2:**
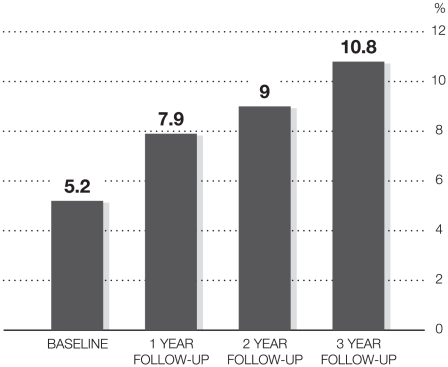
Percentage of respondents rating their sleep quality as pretty poor or poor.

In all datasets, sleep quality was highest at baseline when all participants were still students (Table 1). Furthermore, the percentage of respondents who rated their sleep quality as pretty poor or poor increased on each and every measurement occasion (Figure 2).

**Table 1:** Summary of mean values of sleep quality in the four datasets.

Dataset	Sleep quality_t1_Mean (SD)	Sleep quality_t2_Mean (SD)	Sleep quality_t3_Mean (SD)	Sleep quality_t4_Mean (SD)	n
**Complete dataset**	4.26 (.88)	4.10 (.93)	4.05 (.99)	4.02 (1.04)	1114
**The listwise sample (Two-way ANCOVA)**	4.28 (.86)	4.14 (.90)	4.08 (.96)	4.04 (1.03)	846
**EM-imputed**	4.26 (.88)	4.10 (.91)	4.03 (.96)	4.00 (.98)	1114
**Working as a nurse at all three follow-ups**	4.25 (.89)	4.17 (.90)	4.16 (.94)	4.15 (.98)	554

### Repeated measures ANOVA and ANCOVA in the listwise sample

**Figure 3:**
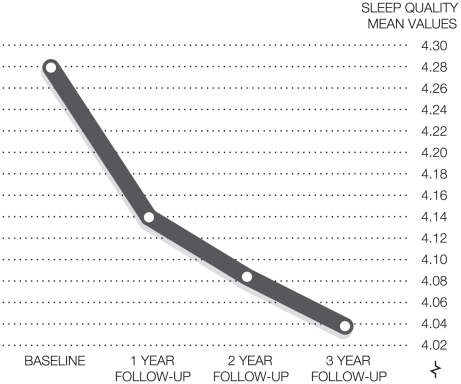
Repeated measures ANOVA illustrating the deterioration in sleep quality mean values over time. (n = 846).

The repeated measure ANOVA indicates a general significant decrease in sleep quality over time (Figure 3, time effect: F = 19.147_df = 3_, p<.0001, eta sq = .022, t3–t4 is ns). The most pronounced decline in sleep quality for the whole group occurred immediately after the transition from study life to working life. There was no significant change between t3–t4, although the change between t2–t3 was significant (p<.05).

There were statistically significant sex (t = −2.06_df = 1_, p<.05) and age (F = 5.731_df = 2_, p = .001) related differences at baseline, where men (mean  = 4.42±.78) rated better sleep quality than women (mean  = 4.24±.90) and participants older than 29 (29–36 years mean  = 4.13±.95; >36 years mean  = 4.18±.95) rated worse sleep quality than those younger than 29 (24–28 years mean  = 4.34±.82; <24 years mean  = 4.41±.78). However, the two-way ANCOVA that adjusts for possible initial differences showed no significant interaction effects between the four age groups over time. There were no differences in sleep quality decline between women and men, even if there was a tendency (p = .079) for men to decline more immediately after the transition but have better recovery in the subsequent follow-ups. Furthermore, there were no differences in sleep quality decline regardless of whether the participants at baseline had partners, were living alone (or with parents), living with children at home, had previous experience from work within healthcare, or whether they felt their education had prepared them well enough to work as nurses. However, the nurses who had previous nursing assistant training exhibited marginally but consistently better sleep quality over time compared to those that did not (time effect: F = 84.691_df = 3_, p<.0001, eta sq  = .092; time x group effect: F = 2.851_df = 3_, p<.05, eta sq  = .003).

Consistent with the listwise sample, the two-way ANCOVA for the sample that included participants working as a nurse at every follow-up and in the EM-imputed sample showed no significant effects between age groups or sexes over time. Similarly, there were no differences in sleep quality decline, regardless of whether the participants at baseline had partners, were living alone (or with parents), living with children at home, or whether they had previous experience of work within healthcare, or felt their education had prepared them well enough to work as nurses. However, the nurses who had previous nursing assistant training exhibited marginally but consistently better sleep quality over time compared to those that did not in both the “working as a nurse” sample (time effect: F = 64.233_df = 3_, p<.0001, eta sq  = .105; time x group effect: F = 3.817_df = 3_, p = .01, eta sq  = .007) and in the EM-imputed sample (time effect: F = 120.798_df = 3_, p<.0001, eta sq  = .099; time x group effect: F = 2.880_df = 3_, p<.05, eta sq  = .003).

## Discussion

The aim of the present study was to longitudinally monitor the development of sleep quality in nurses, starting from the last semester in higher education with subsequent follow-ups in working life. The results imply a continuous decline in sleep quality among nurses during the three years of follow-up. The most pronounced short-term decline in sleep quality seems to occur in the transition between student life and working life. Thereafter, a less sharp decline in sleep quality occurs over the subsequent follow-ups. Over time, there were no differences between women and men or between different age groups. Only a modest difference was found for those who had previously worked as nursing assistants. They exhibited marginally, but consistently better sleep quality over time compared to those who did not share that work experience. With regard to poor sleep quality, the prevalence doubled (5.2%–10.8%) from the first to fourth assessment.

With regard to generalizability of the results, this longitudinal study may of course suffer from problems of wave dropout. However, neither sleep quality levels nor age and sex predicted future participation on any measurement occasion. Consequently, the dropout rate, which ranged between 8–16% across the follow-ups, should not influence the generalizability of the results.

Overall, the effect sizes for the decline in sleep quality were rather small [57], with eta squares ranging from 0.003–0.102. However, in spite of the statistically, relatively small decline, the trend is negative over time with a consistent pattern of generally worsening sleep quality or insufficient recovery. Since it often takes years to develop a significant sleep problem [58], the results of the present study could indicate the preliminary stages of forthcoming sleep disorders in this group. Indeed, other studies have found that fatigue due to inadequate or impaired sleep is a common problem among nurses [4], [22], [23], [24], [29]. Possible explanations of impaired sleep among nurses could be repeated exposure to psychosocial stress in combination with irregular work hours, long shifts and or shift work, other work environmental risk factors, and work/family interferences [4], [18], [20], [21], [22], [26], [30], [31], [36], [38], [59], [60].

Previous studies estimate the prevalence of poor sleep in the general population to be between 10–30% [34], [36], [37], [38], [39]. Considering this, it may be argued that the relatively low prevalence of poor sleep found in the present study is not very alarming. Indeed, the mean level of sleep quality in the total sample is high, relative to the general population prevalence figures. From another perspective, however, the deterioration in sleep quality may indicate (for reasons such as stress, work environment, work/family interferences, age) malfunctioning recovery processes in some individuals. The immediate sharp decline in sleep quality after the transition from nursing education to working life should in ideal circumstances, recover after the initial “reality shock” of becoming a nurse. For some reason though, sleep quality ratings continue to decline, even if the decline is less pronounced over time. This may further strengthen the hypothesis that recovery processes increasingly malfunction for some reason, once nurses enter working life. On a long-term basis, the disturbed sleep and insufficient recovery, combined with other factors such as stress, may increase the vulnerability to or induce stress-related diseases and disorders.

Thus, the present study findings of continuously declining sleep quality may well indicate increased stress among nurses. Previous studies have found, for instance, that the transition from nursing education to working life is particularly stressful [46], [47], [48]. Similarly, our results show that decline in sleep quality was largest immediately after the transition. Subsequent decline was more modest. Behind the assumption that increased stress may explain declining sleep quality is the large body of evidence indicating the negative effects of stress on sleep [18], [24], [32], [34], [36], [38], [42], [43]. Furthermore, previous studies report that the nursing profession often includes several sources of psychosocial and physical stress, such as responsibility and accountability (sometimes in relation to ethical considerations), heavy lifting, repetitive work and a high physical work load, and necessitates dealing with socially delicate situations involving conflict of loyalties, bureaucratic pressures, and a lack of self-confidence [46], [47], [51]. However, the transition from a more flexible student life to a structured and perhaps more demanding working one poses a new kind of distress with regard to relationships and family routines. Thus, the possible “spill-over” effects from either family, working life or both, are difficult to disentangle and may on a longer term basis be a burden.

Irrespective of the sources of strain, high levels of stress and fatigue among nurses may threaten patient safety as well as the nurses' own health [4], [10], [22], [25], [26], [27], [28], [60], [61]. Consequently, the results of the present study, and similar findings in previous research, raise questions as to how a sustainable working life for nurses can accomplished. There is clearly a need for stress-management (including sleep management) interventions among nurses. With regard to patient safety and nurses' health issues, it is advised that such interventions be proactively implemented both in nursing education as well as in healthcare organizations. For the nursing students, special emphasis can be given to helping them handle the transition between nursing education and working life. Such stress-management interventions might better prepare the students for their professional life as nurses. Furthermore, prolonged supervision and acclimatization procedures during the first year could prevent some of the stress of the transition from nursing education to working life. If stress-management interventions are targeted at healthcare on both an educational and professional level, this might better prepare nurses for working life and also prevent and/or reduce the risk of their making future medical errors or experiencing ill health in the future. Stress-management interventions should preferably be provided on both an individual and organizational level.

In order to determine risk factors for and understand the underlying mechanisms of declining sleep quality among nurses, a more thorough examination of possible predictors of various sleep quality outcomes is needed. It would be valuable to determine characteristics among nurses that may predict various trends such as maintenance/improvement or fluctuation and deterioration in sleep quality over time. Such predictors could be used to explore individual differences in intra-individual change over time and help uncover possible individual change patterns that may be hidden in the present group analyses [56].

### Strengths and limitations

One strength with the present sample is the good participation rate and that potential forms of selection bias have been dismissed. This means that the sample is largely representative of the population in terms of age and gender. However, a potential source of bias is the educational environment as for instance different organizational climates and locations may have an impact on stress and sleep. Also, it is not completely certain that the observed effects (and the need for counter-measures) are exclusive to nurses; inclusion of a non-nursing control group could have rendered important information about causality.

### Conclusions

This study found a small, but continuous decline in sleep quality among nurses during the three years of follow-up, starting from their last semester of nursing education and continuing for three years into their working life. The most pronounced decline in sleep quality seems to occur in the transition between student life and working life and there are no age- or sex-related differences.
